# A novel T-cell epitope in the transmembrane region of the hepatitis B virus envelope protein responds upon dendritic cell expansion

**DOI:** 10.1007/s00705-018-4095-0

**Published:** 2018-11-10

**Authors:** Lubiao Chen, Ying Zhang, Shaoquan Zhang, Youming Chen, Xin Shu, Jing Lai, Hong Cao, Yifan Lian, Zania Stamataki, Yuehua Huang

**Affiliations:** 1 Department of Infectious Diseases, The Third Affiliated Hospital of Sun Yat-sen University, 600 Tianhe Road, Guangzhou, 510630 China; 2 Guangdong Provincial Key Laboratory of Liver Disease Research, The Third Affiliated Hospital of Sun Yat-sen University, Guangzhou, China; 3 National Institute for Health Research Birmingham Liver Biomedical Research Unit, Institute of Immunology and Immunotherapy, University of Birmingham, Birmingham, UK

## Abstract

Restoring antiviral immunity is a promising immunotherapeutic approach to the treatment of chronic hepatitis B virus (HBV) infection. Dendritic cells play a crucial role in triggering antiviral immunity. In this study, we identified immunodominant epitopes prevalent in CD8^+^ T cell responses. We characterized the hierarchy of HBV epitopes targeted by CD8^+^ T cells following autologous monocyte-derived dendritic cell (moDC) expansion in HBV-infected subjects with distinct disease stages: treatment-naïve (TN group, n = 168), treatment with complete virological response (TR group, n = 72), and resolved HBV infection (RS group, n = 28). T cell responses against 32 HBV epitopes were measured upon moDC expansion. Several subdominant epitopes that triggered HBV-specific CD8^+^ T cell responses were identified. These epitopes’ responses varied in individuals with different disease stages. Moreover, the most immunodominant and immunoprevalent epitope included the envelope residues 256-270 (Env_256-270_), corresponding to amino acid residues 93-107 in the small HBV surface protein (SHBs) across three patient groups. The frequency of Env_256-270_-specific interferon-γ-producing T cells was the highest in the RS group and the lowest in the TN group. In addition, individuals with HLA-A*02:03/02:06/02:07 were capable of responding to Env_256-270_. Env_256-270_-specific CD8^+^ T cells tolerated amino acid variations within the epitope detected in HBV genotypes B and C. This suggests that Env_256-270_ in SHBs is crucial in HBV-specific T cell immunity following autologous moDC expansion. It might be a potential target epitope for dendritic-cell-based immunotherapy for CHB patients with complete viral suppression by long-term NAs treatment.

**Electronic supplementary material:**

The online version of this article (10.1007/s00705-018-4095-0) contains supplementary material, which is available to authorized users.

## Introduction

Over 240 million people worldwide are chronically infected with hepatitis B virus (HBV), resulting in about 1 million deaths per year due to liver failure or liver cancer [1]. Interferon (IFN) and nucleot(s)ide analogues (NAs) are currently approved for antiviral treatment of chronic HBV infection. IFN has many side effects, and NAs require life-long use. Moreover, even the most potent antiviral agents cannot eliminate the risk of liver cancer [2], and the combination of NAs does not completely eliminate the virus [3, 4]. Thus, there remains an urgent need for novel therapies for this disease.

Immunotherapy has demonstrated some clinical effectiveness in tumors that are associated with an inflammatory or immune response, such as liver cancer, melanoma, and renal cell carcinoma [5–7]. It has also shown effects on chronic viral infection, including chronic hepatitis B (CHB) [8]. HBV replicates non-cytopathically in hepatocytes, and the virus-related diseases are attributed to chronic immune-mediated inflammatory events [9]. An inflammatory liver associated with HBV infection possesses characteristics that render it a potential target for immunotherapeutic manipulation. For example, lymphocytes are actively recruited to the infected liver [10], and their specific mechanisms to recognize and induce the death of infected hepatocytes suggest the potential for cytotoxic effector cell activation [11]. In addition, circulating lymphocytes derived from CHB display antiviral activity after expanding with HBV peptides *ex vivo* [12]. However, these virus-specific lymphocytes in CHB patients are only partially activated and proliferate only at very low levels, suggesting that immunosuppressive mechanisms prevent T cells from maturing into antiviral effector cells [13].

Dendritic cells (DCs) are the most potent professional antigen-presenting cells (APCs) that can capture, process, and present antigens to naive T cells, thereby stimulating their proliferation and activation [14, 15]. They provide the optimal co-stimulatory environment, with high levels of major histocompatibility complex (MHC) class I and class II co-stimulatory molecules, adhesion molecules, and stimulatory cytokines to evoke an immunostimulatory signal against the antigen [16]. DC-based immunotherapy has been tested in clinical trials in melanoma, prostate cancer, and hepatocellular carcinoma [17–20]. Currently, *in vitro-*cultured monocyte-derived DCs (moDCs) loaded with recombinant antigens have been used to vaccinate CHB patients [21, 22]. These studies show that DC vaccines effectively restore immunity and improve viral control, serological response, and biochemical normalization in some CHB patients. Safety with variable efficacy has been demonstrated.

Although the potential efficacy of DC vaccine immunotherapy has been demonstrated in CHB patients, results vary among patients with different disease phases. It is unclear whether viral factors affect the antiviral efficacy of the DC vaccine. More importantly, there have been only a limited number of studies investigating the prevalence and dominance of HBV-specific T cell responses against different viral epitopes in moDC expansion. It is also unknown how the vertical immunodominance of these HBV-specific T cells changes after moDC expansion in the same CHB individuals before and after antiviral therapy. The aim of this study was to compare patients with chronic HBV infection to those with resolved infection in terms of their HBV-specific T cell repertoire following moDC expansion. We also set out to identify the immunodominant HBV epitopes recognized by HBV-specific T cells in CHB after moDC *in vitro* expansion.

## Materials and methods

### Study subjects

This study was conducted on 268 individuals, including 168 CHB-treatment-naive patients who were HBeAg positive (TN group), 72 CHB-NA-treatment responders (including 57 patients who received entecavir and 15 patients who received telbivudine) with complete suppression of HBV replication (HBV DNA < 20 IU/ml) for at least one year and HBeAg-negative status but sustained HBsAg-positive status (TR group), and 28 patients with resolved HBV infection (including 18 who received pegylated IFN (Peg-IFN) therapy and 10 who spontaneously resolved an acute hepatitis B infection) and HBsAg seroconversion within two months (RS group). Twenty healthy subjects (HBsAg, anti-HBs, HBeAg, anti-HBe and anti-HBc negative) served as healthy controls (HC group). Another nine CHB patients who have been on tenofovir disoproxil fumarate (TDF) treatment for two years (96 weeks) were also included. All subjects were enrolled at the Department of Infectious Diseases of the Third Affiliated Hospital of Sun Yat-sen University from January 2013 to July 2016. Patients who were coinfected with human immunodeficiency virus, hepatitis C virus, or hepatitis D virus or had been treated with immunosuppressive drugs for other diseases were excluded.

Time points for blood sample collection were as follows: i) during the first visit for the TN group, ii) after one year of NA antiviral treatment for the TR group, iii) at the 24th week after HBsAg clearance for the RS group. Unfortunately, serial blood samples were not collected at baseline or other time points during antiviral treatment for the TR and RS groups.

- **Clinical, virological and serological parameters** (see Supplementary Materials).
- **PBMC isolation and flow cytometry sorting** (see Supplementary Materials).
- **HBsAg-pulsed PBMCs or autologous moDCs expansion** (see Supplementary Materials).
- **Autologous moDC phenotyping** (see Supplementary Materials).

### Synthetic peptides and expansion of HBV-specific T cells by autologous moDCs

A library of 313 synthetic 15-mer peptides overlapping by 10 amino acid residues covering the whole HBV genotype B and C proteome sequences, was purchased from Mimotopes Pty Ltd (Clayton, Victoria, Australia). The pools of core and X peptides were made into a 9-by-8 matrix containing eight or nine peptides per pool. Envelope peptides were pooled in a 9-by-9 matrix containing nine peptides per pool. Polymerase peptides formed a 14-by-12 matrix containing 12 or 14 peptides per pool [23]. For expansion of HBV-specific T cells, large panels of all 313 synthetic peptides were pooled in four mixtures covering the entire core, envelope, X, and polymerase proteins of different HBV genotypes. On day 6, the autologous PBMCs were thawed and recovered overnight in RPMI 1640 medium containing 10% Gibco fetal bovine serum (Thermo Fisher Scientific, MA, USA). Then, these cell populations were mixed with autologous moDCs at a 10:1 (lymphocyte/DC) ratio. The cells were incubated for 10 days in Aim-V + 2% human AB serum + IL-2 (20 U/ml). After 10 days of incubation, T cell responses were tested using IFN-γ enzyme-linked immunospot (ELISPOT) assay with the incubation of overlapping viral peptides for 18 hours as described previously [24]. A positive response for moDC-expanded T cells was defined as more than two times the mean of spots in unstimulated wells or more than 10 spots per 1 × 10^5^ cells. Whenever enough cells were available, intracellular cytokine staining (ICS) for IFN-γ by flow cytometry analysis was used to confirm positive responses to moDC expansion by ELISPOT assay.

### Peptide-HLA class I tetramers

Phycoerythrin (PE)-labeled tetrameric synthetic peptide-HLA class I complexes representing HLA-A2-restricted epitopes within the core, envelope, and polymerase proteins of HBV genotype B and C were purchased from Proimmune (Oxford, UK). The amino acid sequences of these HBV tetramers (HBV-TET) are shown in Supplementary Table 1. For immunophenotyping, PBMCs were incubated for 15 minutes at room temperature with individual core or polymerase tetramers (Core_18-27_ or Pol_453-461_) or with a pool of envelope tetramers (Env_194-202,_ Env_346-354_, Env_349-358_ and Env_359-368_). Fluorescent antibodies were then added, and incubation was continued for 15 minutes. After washing with PBS containing 0.1% fetal calf serum, HBV-specific CD8^+^ T cells were immediately sorted on a FACSCanto flow cytometer (Becton Dickinson, CA, USA) and analyzed using BD FACSDiva software (Becton Dickinson, CA, USA). Then, ICS for IFN-γ in HBV-TET^+^ CD8^+^ T cells was carried out to confirm the response to moDC expansion as described below.

### Intracellular cytokine staining for IFN-γ

*In vitro*-expanded peripheral blood mononuclear cells (PBMCs) plus moDCs were incubated in medium alone (control) or with viral peptides (5 μg/ml) for 1 hour, brefeldin A (10 mg/ml) was added, and incubation was continued for an additional 4 hours. The cells were washed and stained with anti-CD8-PE-Cy7 and anti-CD3-PerCp-Cy5.5 monoclonal antibodies (BD Bioscience-Pharmingen, CA, USA) for 30 minutes at 4°C and then were fixed and permeabilized using Cytofix/Cytoperm Fixation/Permeabilization solution (BD Biosciences, CA, USA) according to the manufacturer’s instructions. Cells were stained with anti-IFN-γ-V450 (BD Biosciences, CA, USA) for 30 minutes on ice, washed, and then analyzed by flow cytometry as described elsewhere [25].

### Statistical analysis

The Mann-Whitney U test and Wilcoxon paired rank sum test were used to compare the parameters between two unpaired and paired groups, respectively. The Kruskal-Wallis H test was conducted for multiple comparisons. The Pearson chi-square test or Fisher’s exact test was applied to analyze the enumeration data. Data were expressed as a frequency (percentage), mean ± standard error (SE) or median (interquartile range/IQR) as appropriate. Association between two continuous variables was determined using the Pearson correlation coefficient and a linear regression model. All statistical tests were performed using SPSS 22.0 (SPSS Inc. Chicago, IL, USA). A *P*-value less than 0.05 was considered statistically significant.

## Results

### Generation of autologous moDCs and phenotyping in HBV-infected subjects

We studied 268 HBV-infected individuals, including 168 in the TN group, 72 in the TR group, and 28 in the RS group. Another 20 healthy subjects served as healthy controls (HC group). The clinical characteristics of the patients are shown in Table 1. Cell-surface markers in the moDC population, including MHC-I, CD11c, CD80, CD83 and CD86, were analyzed. The frequencies of these markers did not show any significant differences among these four groups (Supplementary Fig. 1). These data indicated that these autologous moDCs have a similar ability to differentiate regardless of disease stages in HBV infection, and moDC differentiation in the different HBV-infected groups was comparable to that in the uninfected HC group.

**Table 1:** Characteristics of the study populations

Characteristics	TN (n = 168)	TR (n = 72)	RS (n = 28)	*P* value		
				TN *vs.*TR	TN *vs.* RS	TR *vs.* RS
Male gender^a^	108 (64%)	51 (71%)	21 (75%)	NS	NS	NS
Age (years)^b^	28 (19-37)	38(29-48)	30 (21-38)	NS	NS	NS
HBeAg (+)^a^	168(100%)	0 (0%)	0 (0%)	< 0.0001*	< 0.0001*	NA
Anti-HBe (+)^a^	0 (0%)	51(71%)	28 (100%)	< 0.0001*	< 0.0001*	0.0013*
ALT (U/L)^b^	47 (17-170)	32 (25-40)	29(20-38)	0.0420*	0.0336*	NS
HBV DNA (log_10_ IU/ml)^b^	7.3(4.6-8.9)	ND	ND	< 0.0001*	< 0.0001*	NA
HBsAg (log_10_ IU/ml)^b^	4.7(3.0-5.1)	3.7(2.4-4.5)	ND	0.0330*	< 0.0001*	< 0.0001*
Genotypes^a^						
B	114 (68%)	40 (56%)	15 (54%)	NS	NS	NS
C	54 (32%)	31 (43%)	8 (29%)	–	–	–
Others	0 (0%)	1 (1%)	5 (18%)	–	–	–
FibroScan (kPa)^b^	6.3 (4.5-8.8)	6.2 (5.6-8.8)	5.2(4.2-5.8)	NS	NS	NS

Abbreviations: HBeAg, hepatitis B e antigen; anti-HBe, hepatitis B e antibody; ALT, alanine aminotransferase; HBV, hepatitis B virus; HBsAg, hepatitis B surface antigen. ND, not detectable (HBsAg < 0.05 IU/ml; HBV DNA < 20 IU/ml); NA, not applicable; NS, not significant (*P* ≥ 0.05)

*Statistically significant (*P* < 0.05)

^a^Data were expressed as frequency (percentage)

^b^Data were expressed as median (interquartile range)

### Induction of HBV-specific CD8^+^ T cells by autologous moDCs to produce IFN-γ

**Fig. 1:**
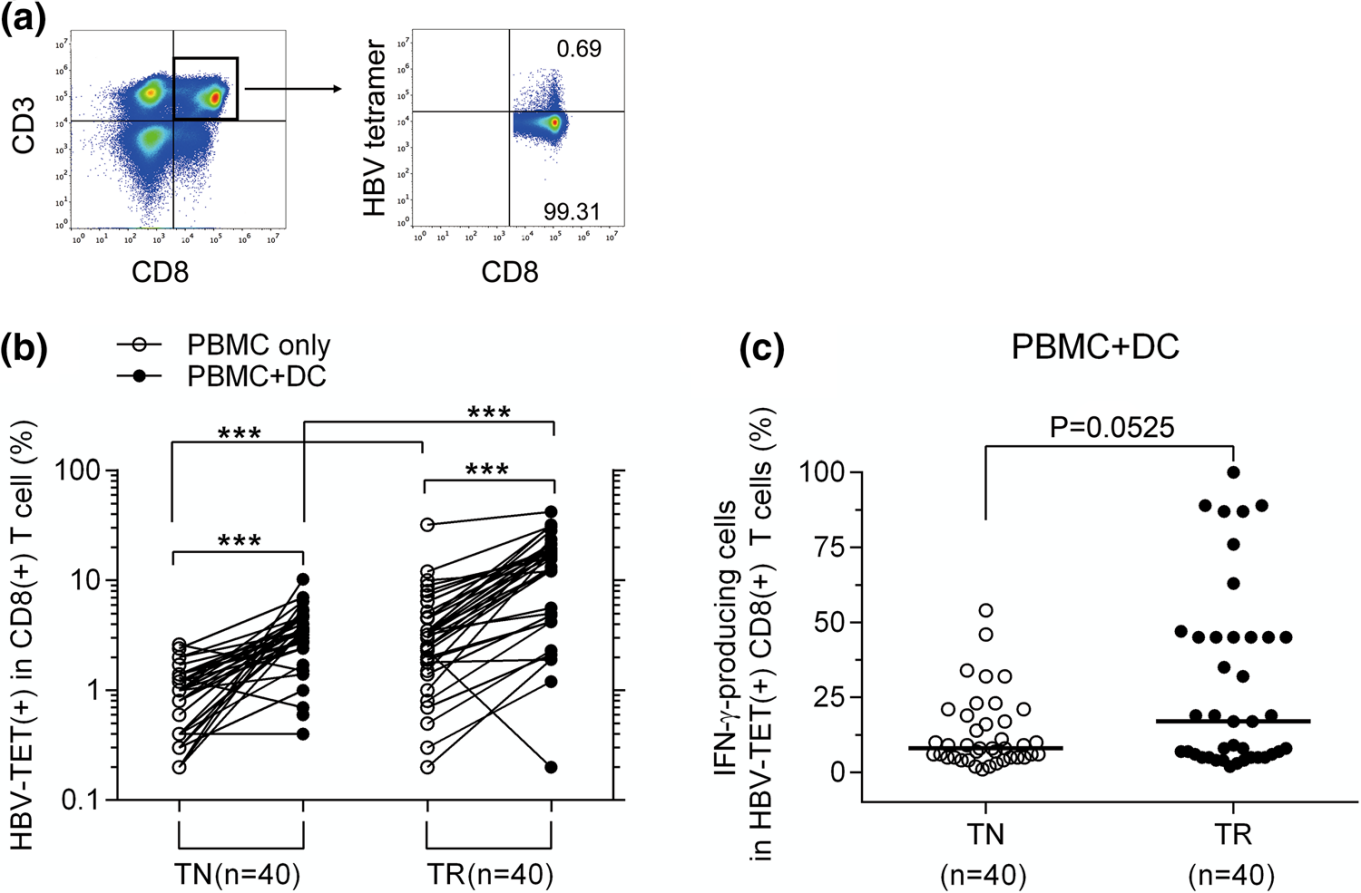
Frequency of HBV-specific T cells upon expansion by PBMC only or PBMC plus moDC (PBMC + DC) in CHB patients. (**a)** Representative example of HBV-specific CD8^+^ T cells after expansion with moDCs *ex vivo* (by FCS with HBV-TET). (**b)** Percentages of HBV-TET^+^ HBV-specific cells in total CD8+ T cells after expansion with PBMC only or PBMC + DC in the TN and TR groups. (**c)** Percentages of IFN-γ-producing cells in HBV-TET^+^ HBV-specific CD8^+^ T cells after expansion with PBMC + DC in the TN and TR groups. FCS with HBV-TET (**a, b**) or ICS (**c**) was performed to measure the frequency of HBV-specific T cells. Differences within the TN or the TR group (**b**) were assessed by the Wilcoxon paired rank sum test. Differences between the TN and TR groups (**b, c**) were analyzed by the Mann-Whitney U test. ^***^, *P* < 0.0001. HBV, hepatitis B virus; PBMC, peripheral blood mononuclear cell; moDCs, monocyte-derived dendritic cells; DC, dendritic cell; CHB, chronic hepatitis B; FCS, flow cytometry sorting; HBV-TET, HBV tetramer; IFN-γ, interferon-gamma; ICS, intracellular cytokine staining

A previous study showed robust activation of DCs via T-cell-receptor-redirected CD8^+^ T cells upon their differentiation in CHB patients [12]. We examined whether mature HBsAg-pulsed moDCs could also be used to expand autologous HBV-specific T cells in CHB by testing 80 patients (40 from the TN group and 40 from the TR group) (Supplementary Table 2). A pool of all six HLA-A2-restricted tetramers (Supplementary Table 1) was used to assess the frequency of HBV-specific CD8^+^ T cells from PBMCs directly (PBMC only) or PBMCs after expansion for 10 days with HBsAg-pulsed moDCs (PBMC+DC). Unlike the dysfunctional/deleted HBV-specific T cell responses observed in chronic HBV infection [23], our data revealed that HBV-specific CD8^+^ T cells were significantly enhanced by moDC antigen presentation as detected by flow cytometry with HBV-TET staining (Fig. 1a). The percentage of HBV-TET^+^ CD8^+^ T cells expanded by PBMC + DC was about 3.9-fold and 4.6-fold greater than that by PBMC only in the TN (median: 3.5% *vs.* 0.9%, *P* < 0.0001) and TR (median: 14.4% *vs.* 3.1%, *P* < 0.0001) group, respectively (Fig. 1b). More importantly, the difference in the proportion of IFN-γ-producing cells in HBV-TET^+^ CD8^+^ T cells measured by ICS indicated borderline significance between these two groups (median: 17.0% *vs.* 8.0%, *P* = 0.0525) (Fig. 1c). These findings imply that antiviral therapy may increase the number of DC-expanded HBV-specific CD8^+^ T cells in CHB patients.

**Fig. 2:**
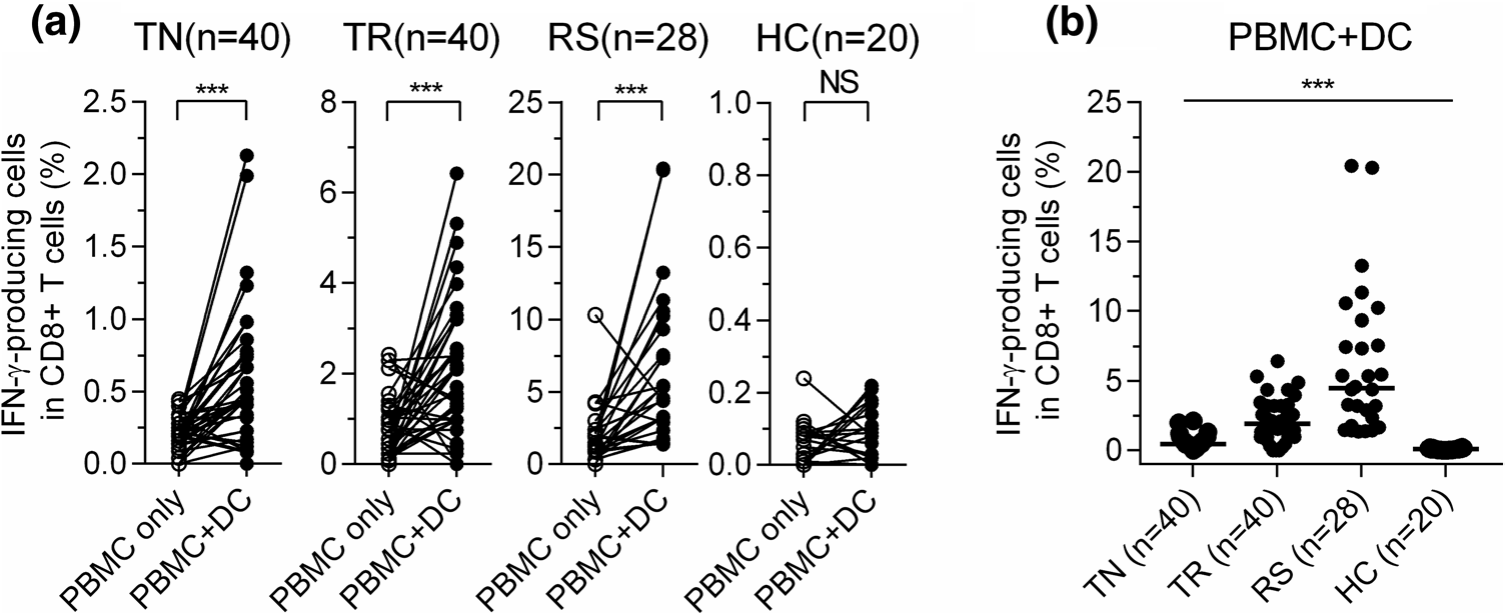
Responses of HBV-specific T cells expanded by PBMC only or PBMC plus moDC (PBMC + DC). (**a)** Percentages of IFN-γ-producing cells among the total CD8^+^ T cells expanded by PBMC + DC were much higher than those by PBMC only in the TN, TR and RS groups, but not in the HC group. (**b)** The percentage of IFN-γ-producing cells among the total CD8^+^ T cells expanded by PBMC + DC was the highest in the RS group and the lowest in the HC group (differences between any two groups are significant). The frequency of HBV-specific CD8^+^ T cells was measured by FCS after ICS for IFN-γ. Wilcoxon paired rank sum test (**a**). Mann-Whitney U test (**b**). ^***^, *P* < 0.0001. NS, not significant; PBMC, peripheral blood mononuclear cell; moDCs, monocyte-derived dendritic cells; DC, dendritic cell; IFN-γ, interferon-gamma; ICS, intracellular cytokine staining

IFN-γ produced by HBV-specific CD8^+^ T cells was measured by ICS using the pool of all 313 overlapping peptides as the stimulant in these 80 patients, 28 subjects from the RS group and 20 subjects from the HC group. The percentage of IFN-γ-producing cells in total CD8^+^ T cells was significantly increased after expansion by PBMC + DC compared to PBMC only across the TN, TR, and RS groups, but it was not in the HC group (Fig. 2a). Better responses to autologous moDC expansion were observed in the RS and TR groups than in the TN and HC groups. The percentage of detectable IFN-γ-producing cells in total CD8^+^ T cells was the highest in the RS group and the lowest in the HC group (medians: 4.48% in RS, 1.91% in TR, 0.45% in TN, 0.09% in HC). The difference between each pair of groups was statistically significant (*P* < 0.0001) (Fig. 2b).

### Immunoprevalence and immunodominance of the HBV-specific CD8^+^ T cell response to HBV epitopes upon autologous moDC expansion

**Fig. 3:**
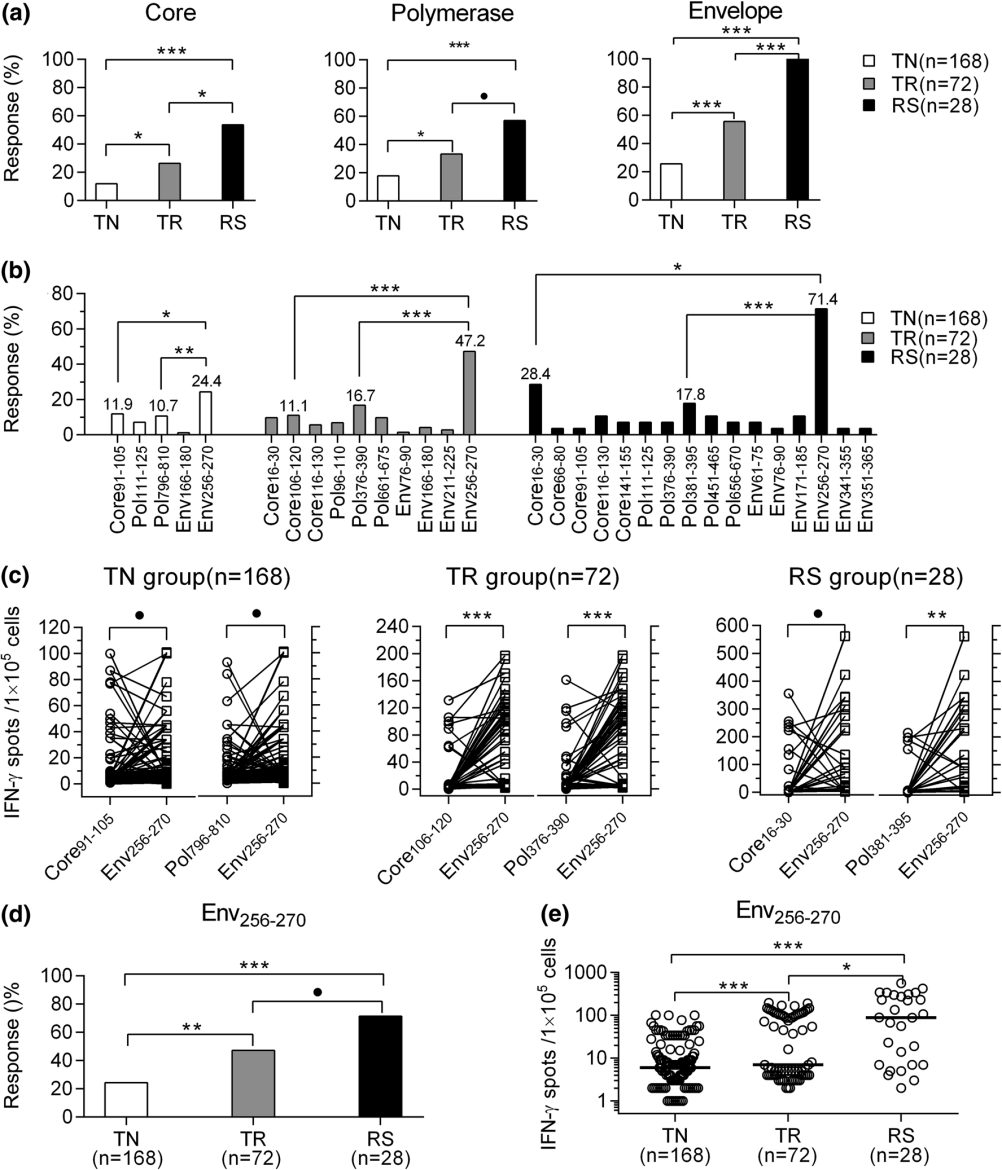
Immunoprevalence and immunodominance of HBV-specific T cell response to HBV epitopes upon autologous moDC expansion. (**a)** The percentage of HBV-specific CD8^+^ T cells against core, polymerase or envelope protein was the highest in the RS group and the lowest in the TN group. (**b)** Env_256-270_ from envelope protein was the most immunoprevalent epitope in the TN, TR and RS groups. (**c)** ELISPOT assay for IFN-γ release indicated that Env_256-270_ was the most immunodominant epitope against HBV by HBV-specific CD8^+^ T cells in the TN, TR or RS group. (**d)** The percentage of response to Env_256-270_ peptide was the highest in the RS group and the lowest in the TN group. (**e)** The frequency of IFN-γ-producing cells against the Env_256-270_ peptide was the highest in the RS group and the lowest in the TN group. A positive response was defined as a frequency of IFN-γ-producing cells more than two times the mean of spots in unstimulated wells or more than 10 spots per 1×10^5^ cells (**a, d**). Panels **d** and **e** are excerpts from panels **b** and **c**. IFN-γ ELISPOT assay was applied to assess the specific T cell response. Pearson chi-square test (**a, b, d**), Wilcoxon paired rank sum test (**c**), Mann-Whitney U test (**e**). ^●^, *P* < 0.05; ^*^, *P* < 0.01; ^**^, *P* < 0.001; ^***^, *P* < 0.0001. HBV, hepatitis B virus; moDC, monocyte-derived dendritic cell; Env_256-270_, envelope residues 256-270; ELISPOT, enzyme-linked immunospot assay; IFN-γ, interferon-gamma

Considering that moDCs from CHB patients stimulated expansion of autologous HBV-specific T cells, we next assessed the response to CD8^+^ T cell epitopes in the current cohort (TN = 168, TR = 72, RS = 28). After *in vitro* moDC expansion for 10 days, HBV-specific responses against the HBV core, envelope, polymerase, and X proteins could be detected by IFN-γ ELISPOT using the corresponding pools of viral peptides as the stimulants, with the weakest response against epitopes in the X protein (data not shown). A total of 20 (11.9%), 19 (26.4%), and 15 (53.6%) subjects showed HBV-specific cytotoxic T cells (CTLs) directed against the core protein in the TN, TR, and RS group, respectively. There were 30 (17.9%), 24 (33.3%), and 16 (57.1%) subjects showing HBV-specific CTLs against polymerase protein, while the envelope protein was recognized by 43 (25.6%), 40 (55.6%), and 28 (100%) subjects in the TN, TR, and RS group, respectively (Fig. 3a).

The discrepancy in the immunoprevalence profile of CD8^+^ T cell responses was observed in patients with different disease stages. Notably, the most prevalent immune response was against residues 256-270 in the envelope protein (Env_256-270_) in three groups (Fig. 3b). Other main prevalent immune epitopes included Core_91-105_ and Pol_796-810_ in the TN group, and Core_106-120_ and Pol_376-390_ in the TR group, Core_16-30_ and Pol_381-395_ in the RS group. These data suggest that HBV-specific CD8^+^ T cells recognize multiple epitopes within the envelope, core, and polymerase proteins during chronic HBV infection upon moDC expansion, while Env_256-270_ is the most prevalent one.

We then evaluated these HBV-specific CD8^+^ T cell immunodominant epitopes separately. After *in vitro* stimulation with these main immunoprevalent peptides with PBMC + DC on CD8^+^ T cells, ELISPOT assay was performed to determine the frequency of IFN-γ-producing cells. We found that Env_256-270_-specific CD8^+^ T cells were dominant over CD8^+^ T cells against epitopes of HBV core protein and polymerase. The level of IFN-γ release elicited by Env_256-270_ was significantly higher than that elicited by the other immunoprevalent epitopes in the TN, TR or RS group (Fig. 3c). These data clearly indicate that the CD8^+^ T cell response against the Env_256-270_ epitope sequence was immunodominant in the majority of HBV-infected subjects in our patient cohort.

Particularly, the proportion of patients recognizing the most common envelope epitope, Env_256-270_, was the highest in the RS group (71.4%), intermediate in the TR group (47.2%), and the lowest in the TN group (24.4%) (Fig. 3d). Similar findings were obtained for IFN-γ release elicited by Env_256-270_ (Fig. 3e).

### Vertical immunodominance of the HBV-specific T cell response

**Fig. 4:**
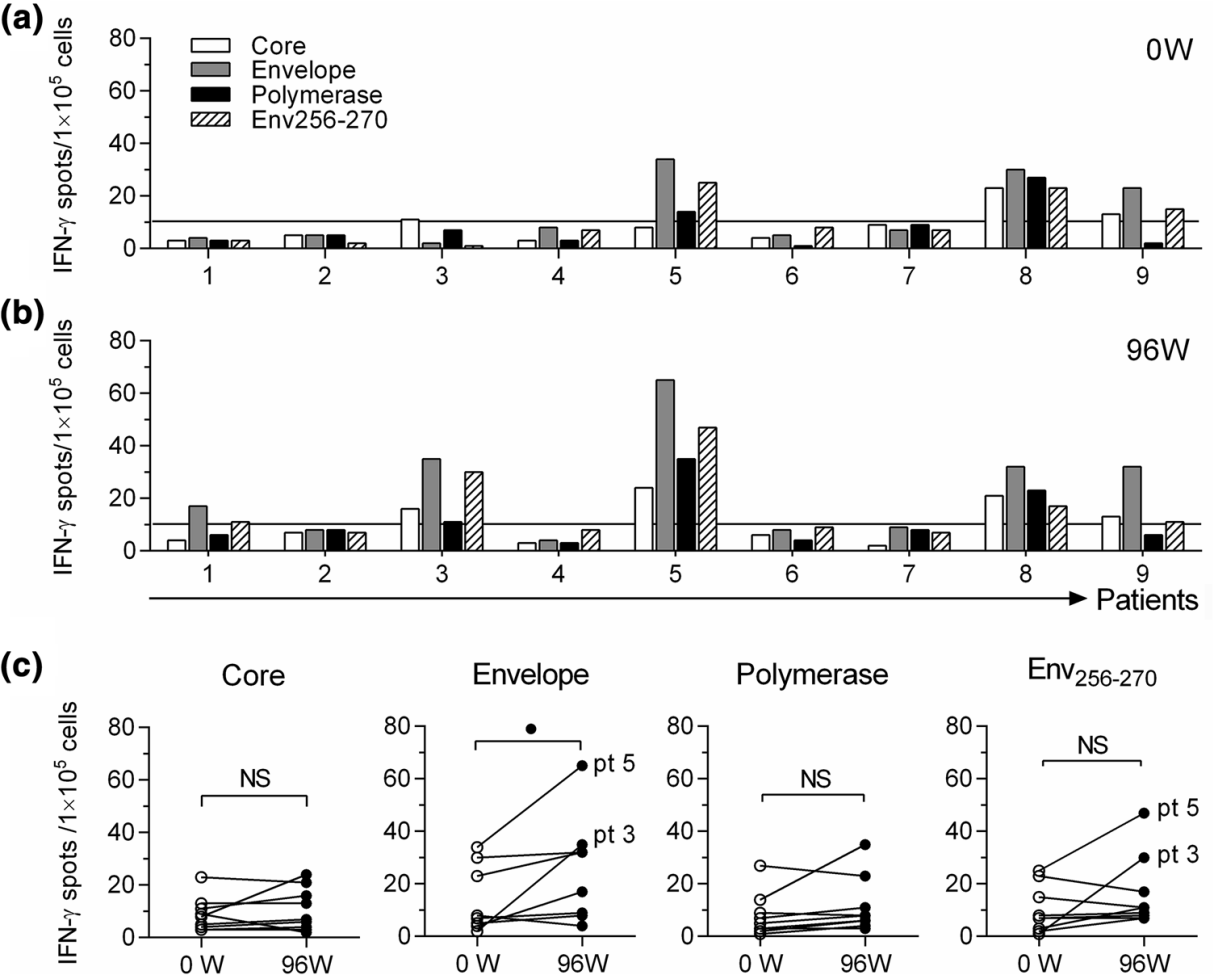
Dynamic changes of vertical immunodominance of HBV-specific T cells in nine CHB patients with TDF antiviral treatment. (**a** and **b**) Responses of HBV-specific T cells to different peptides before (**a**) and after (**b**) 96-weeks of TDF antiviral treatment. (**c)** Comparison of HBV-specific T cell responses in the same 9 patients between 0 and 96 weeks of TDF treatment. IFN-γ ELISPOT assay was applied to assess the specific T cell response. Wilcoxon paired rank sum test (**c**). NS, not significant; ^●^, *P* < 0.05. HBV, hepatitis B virus; CHB, chronic hepatitis B; TDF, tenofovir disoproxil fumarate

We also evaluated the vertical immunodominance of the above major epitopes’ response to HBV-specific T cells. For this purpose, the whole HBV-specific T cell repertoire was studied in nine CHB patients before and after two years of TDF therapy. Patients’ clinical characteristics are listed in Supplementary Table 3. ELISPOT assays were performed after *in vitro* expansion to detect IFN-γ-producing cells. The responses to epitopes of core, envelope, and polymerase after TDF antiviral treatment are shown in Fig. 4. Activated Env_256-270_-specific CD8^+^ T cell responses were seen in five out of nine patients after two years of TDF treatment, compared to three out of nine patients before treatment. A positive response was defined as >10 spots per 1 × 10^5^ cells). The responses of patients 3 and 5 were notably enhanced (Fig. 4c).

### Influence of mutations in Env_256-270_ on the specific CD8^+^ T cell response

Having demonstrated the immunodominance of the Env_256-270_-specific T cell response in CHB, we next analyzed whether amino acid (aa) mutations in this region, corresponding to aa residues 93-107 in the small HBV surface protein (SHBs), can alter T cell recognition. The wild-type (WT) aa sequence *FLLVLLDYQGMLPVC* corresponding to the Env_256-270_ epitope is located within the second transmembrane domain of the S region of the HBV envelope protein (schematic representation in Supplementary Fig. 2).

**Fig. 5:**
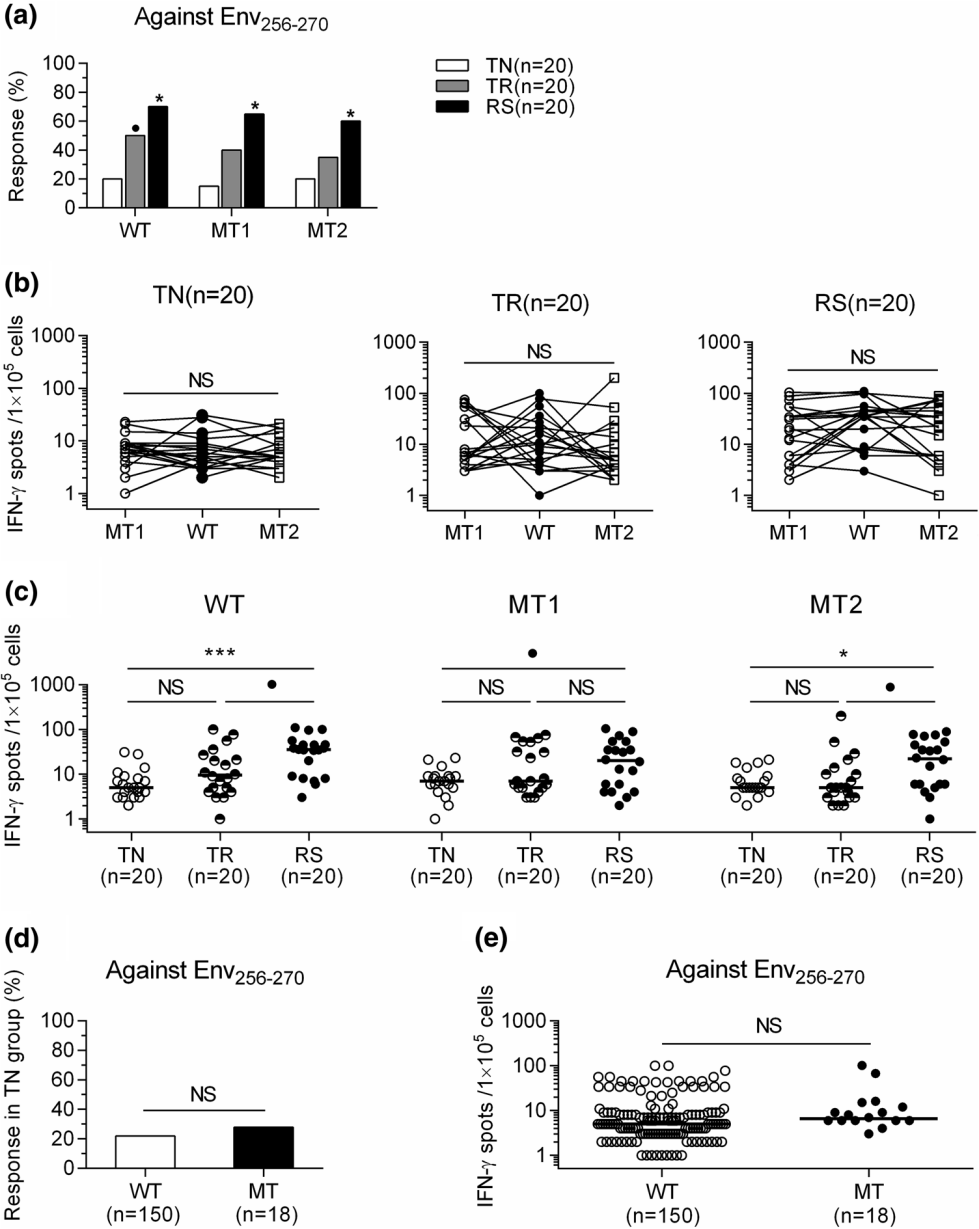
Influence of mutations in the HBV genome on the Env_256-270_-specific T cell response. (**a**) Response to WT, MT1 or MT2 Env_256-270_ peptides among the TN, TR and RS groups (^●^, *P* < 0.05; ^*^, *P* < 0.01 compared to the TN group). The frequency of IFN-γ-producing cells within (**b**) or among (**c**) the TN, TR or RS group against the WT, MT1 or MT2 Env_256-270_ peptide. (**d** and **e**) Percentage of response (**d**) and frequency of IFN-γ-producing cells (**e**) against the Env_256-270_ peptide in the TN group with WT or MT infection. A positive response was defined as a frequency of IFN-γ-producing cells more than two times the mean of spots in unstimulated wells or more than 10 spots per 1×10^5^ cells (**a**). IFN-γ ELISPOT assay was applied to assess the specific T cell response. Fisher’s exact test (**a**), Wilcoxon paired rank sum test (**b**), Mann-Whitney U test (**c, e**), Pearson Chi-square test (**d**). NS, not significant; ^●^, *P* < 0.05; ^*^, *P* < 0.01; ^***^, *P* < 0.0001. Env_256-270_, envelope residues 256-270; WT, wild type; MT, mutated type; MT1, mutant 1 (*L95W*); MT2, mutant 2 (*L98V*); IFN-γ, interferon gamma

A recent study has shown that mutations in this region (at positions 258 and 261, corresponding to 95 and 98 in the SHBs) are able to block S synthesis and subviral particle production [26]. We therefore analyzed whether Env_256-270_-specific CD8^+^ T cells similarly recognized the sequences *FLWVLLDYQGMLPVC* (L95W; mutant 1, MT1) and *FLLVLVDYQGMLPVC* (L98V; mutant 2, MT2) (aa substitutions are underlined), corresponding to the envelope region of residues 256-270 of the HBV genome. Twenty HBV-infected subjects were randomly selected from the TN, TR, and RS groups. Information about these 60 cases is shown in Supplementary Table 4. PBMCs were used to test whether the synthetic mutated type (MT=MT1+MT2) peptides are similar to the WT peptide in their ability to expand Env_256-270_ CD8^+^ T cells *in vitro*. Interestingly, the CD8^+^ T cell response measured by IFN-γ ELISPOT was comparable within the TN, TR or RS group following T cell expansion against synthetic WT, MT1 or MT2 peptide. This demonstrated that the proportion of response against Env_256-270_ with WT, MT1 or MT2 was significantly higher in the RS group than in the TN group (Fig. 5a), but there was borderline significant difference in response against Env_256-270_ with WT between the TR and TN groups. There were no significant differences in the level of IFN-γ release among the TN, TR and RS groups against WT, MT1 or MT2 (Fig. 5b), but it was much higher in the RS group than in the TN or TR group against WT, MT1 or MT2 (Fig. 5c). We also performed sequence analysis on a full-length HBV S gene fragment amplified from 168 serum samples from the TN group, which revealed that eight (4.8%) patients had the L95W mutation, and 10 (6.0%) had the L98V mutation. We then compared the virus-specific response in patients carrying the WT or MT epitope at aa residues 93-107 in the SHBs. As expected, we found comparable proportions of patients with a detectable Env_256-270_-specific CD8^+^ T cell response in patients infected with WT or MT mutations (22.0% *vs.* 27.8%, *P* = 0.5597) (Fig. 5d). The levels of IFN-γ released from patients infected with WT and MT mutations were not significantly different (median: 6.0 *vs*. 6.5, *P* = 0.2127) (Fig. 5e).

Finally, we correlated the frequencies of HBV-specific IFN-γ-producing CD8^+^ T cells with viral loads and antigen titers in 168 untreated CHB patients from the TN group. Univariate analysis showed that higher levels of HBsAg (HR = 2.41, 95% confidence interval [CI]: 1.31-4.20, *P* = 0.0370) and HBV DNA (HR = 2.04, 95% CI: 1.29-3.54, *P* = 0.0412) significantly hindered the production of Env_256-270_-specific IFN-γ. Multivariate analysis showed that the HBsAg titer was the only factor significantly associated with impaired HBV-specific IFN-γ release (HR = 2.72, 95% CI: 1.37-5.11, *P* = 0.0211) (Table 2).

**Table 2:** Univariate and multivariate analyses of virological features correlating with IFN-γ release from Env_256-270_-specific T cells upon DCs differentiation in the TN group (n=168)

Parameters	Univariate analysis HR (95% CI)	*P* value	Multivariate analysis HR (95% CI)	*P* value
HBV DNA level	2.04 (1.29-3.54)	0.0412*	1.42 (0.82-2.47)	0.0892
HBsAg level	2.41 (1.31-4.20)	0.0370*	2.72 (1.37-5.11)	0.0211*
Genotypes	1.26 (0.58-2.64)	0.4504	0.52 (0.15-1.84)	0.2381
*L95M* (MT1)	1.62 (0.68-3.47)	0.5210	1.83 (0.63-4.91)	0.5032
*L98V* (MT2)	1.30 (0.69-2.24)	0.4107	1.36 (0.69-2.71)	0.6118

Abbreviations: HBV, hepatitis B virus; HBsAg, hepatitis B surface antigen. MT1, mutant-1; MT2, mutant-2; 95% CI, 95% confidence interval

*Statistically significant (*P* < 0.05)

### Effect of HBV genotypes and HLA-A2 subtypes on CD8^+^ T cell responses against the Env_256-270_ epitope

**Fig. 6:**
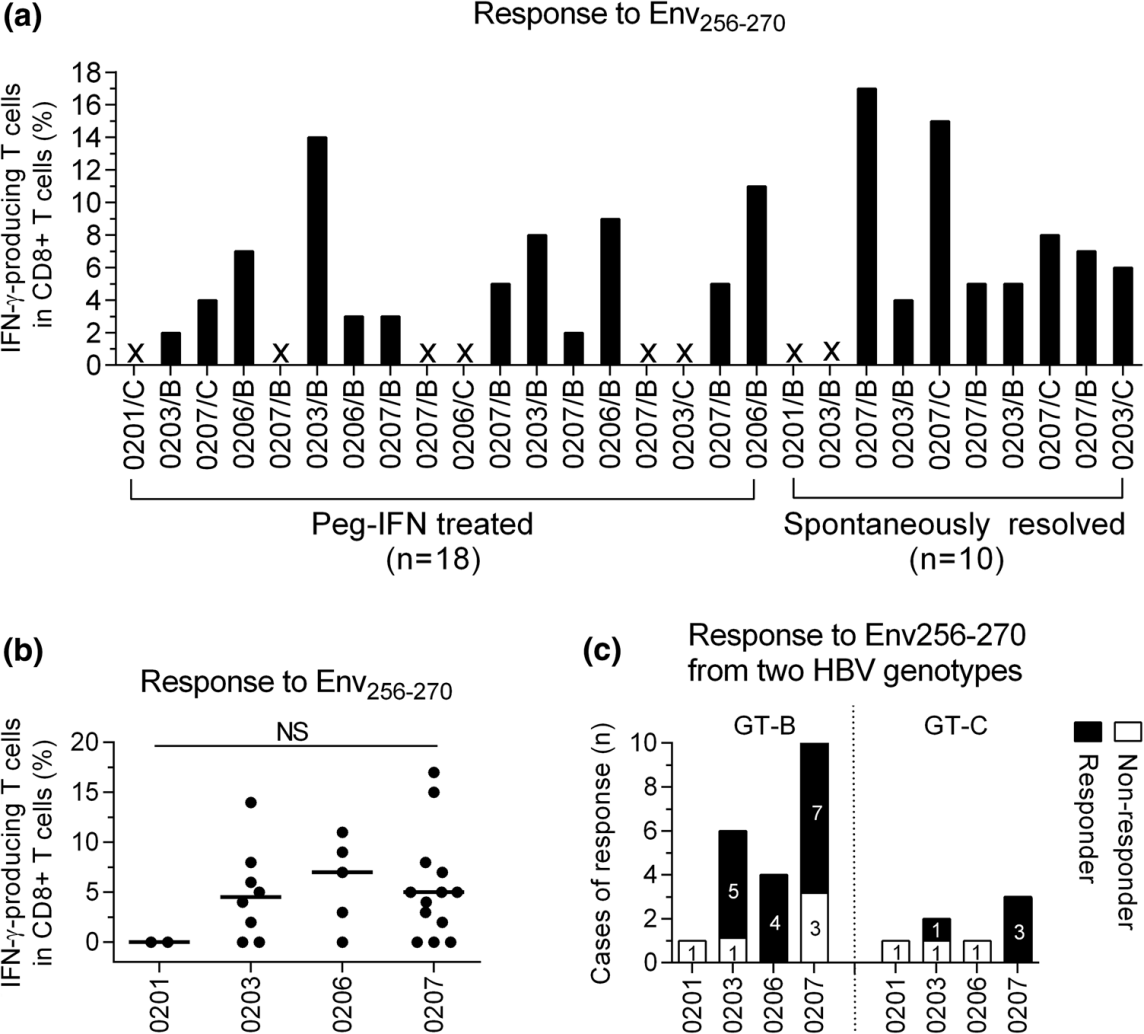
CD8^+^ T cell response against the Env_256-270_ epitope in HLA-A2^+^ subjects from the RS group infected with different HBV genotypes. (**a**) Percentage of IFN-γ-producing T cells among the CD8^+^ T cells against the Env_256-270_ epitope from each subject (labels of x-axis: the number represents the HLA-A2 subtype of each subject and a capital letter represents the HBV genotype. “**×**” indicates no response). (**b**) Percentage of Env_256-270_-specific IFN-γ-producing CD8^+^ T cells in subjects with the indicated HLA-A2 subtypes. (**c**) Frequency of response to the Env_256-270_ epitope in subjects infected with HBV genotype B or C according to different HLA-A2 subtypes (GT-B, genotype B; GT-C, genotype C. The number in the box indicates the corresponding case). Frequency of Env_256-270_-specific CD8^+^ T cells measured by FCS after ICS for IFN-γ. Kruskal-Wallis H test (**b**). NS, not significant; Env_256-270_, envelope residues 256-270; HLA, human leukocyte antigen; HBV, hepatitis B virus; IFN-γ, interferon gamma

A previous study showed that HLA subtypes and HBV genotypes affect the diversity of the HBV-specific CD8^+^ T cell repertoire [23]. Therefore, we investigated whether HLA-A2 subtypes and HBV genotypes can influence CD8^+^ T cell responses against Env_256-270_ in 28 RS subjects. High-resolution HLA-A2 typing was performed on the subjects to determine their HLA-A2^+^ subtypes. The data showed the RS individuals displayed different HLA-A2 subtypes (HLA-A*02:01, n = 2; HLA-A*02:03, n = 8; HLA-A*02:06, n = 5; HLA-A*02:07, n = 13). After *in vitro* moDC expansion for 10 days with the peptides corresponding to the sequence of the HBV genotype, the frequency of the HBV-specific CD8^+^ T cells was determined by ICS for IFN-γ production. The data showed that CD8^+^ T cell responses to the Env_256-270_ epitope were highly influenced by HLA-A2 micropolymorphisms (6/8 of HLA-A*02:03, 4/5 of HLA-A*02:06, and 10/13 of HLA-A*02:07) (Fig. 6a and b). However, none of the HLA-A*02:01 patients responded to this epitope. In addition, there was no significant difference in the response to Env_256-270_ between the subgroups of patients who had received Peg-IFN treatment and those who had spontaneously resolved infection within the RS group (Responders: 12/18 *vs.* 8/10, Fisher’s exact test, *P* = 0.6692) (Fig. 6a). Figure 6c shows the CD8^+^ T cell responses against the Env_256-270_ peptides differing by one amino acid between HBV genotype B and genotype C (Supplementary Table 1). Interestingly, five of the six subjects with of HLA-A*02:03, all four with of HLA-A*02:06, and seven of the ten with of HLA-A*02:07 in the RS group responded to the Env_256-270_ epitope from HBV genotype B, and one of the two with HLA-A*02:03 and all three with HLA-A*02:07 were capable of responding to the Env_256-270_ epitope from HBV genotype C. These results implied that HLA-A2 subtypes and HBV genotypes may influence the Env_256-270_-specific T cell response.

## Discussion

DCs are professional APCs with the ability to take up, process, and present antigen, which in the right context can stimulate both CD4^+^ and CD8^+^ antigen-specific T cell responses. During HBV infection, CD8^+^ T cells target multiple epitopes and establish a hierarchy of dominant and subdominant HBV-specific CD8^+^ T cell responses in the infected subjects. In the present study, we showed that *in vitro* autologous moDC expansion can enhance the stimulatory capacity of HBV-specific T cells in treatment-naive patients, patients receiving antiviral therapy, and individuals with resolved HBV infection. In our CHB patient cohort, the percentage of IFN-γ-producing cells among the HBV-specific CD8^+^ T cells from the TR group was higher than that in the TN group. This difference with borderline significance might be attributed to the small sample sizes of these two groups. The CD8^+^ T cells obtained by moDC expansion were found to target multiple HBV epitopes on the core protein, polymerase and envelope protein. These epitopes’ responses to HBV-specific CD8^+^ T cells varied in individuals at different disease stages. Among the identified epitopes, the most common immunodominant epitope upon autologous moDC expansion was Env_256-270_, corresponding to residues 93-107 in the SHBs. A negative correlation between HBsAg titer and the frequency of HBV-specific IFN-γ-producing CD8^+^ T cells was observed in treatment-naive patients.

HBV antigens are known to have an immune regulatory function in promoting viral persistence through the modulation of the immune response to viruses [27]. Indeed, HBeAg and HBsAg were found here to be crucial viral factors determining patients’ ability to elicit anti-HBV immune responses upon moDC stimulation. The HBV-specific CD8^+^ T cell response progressively increased from untreated HBV patients with a high viral load who were positive for HBeAg to patients with completely depressed HBV DNA replication who were negative for HBeAg, and reached highest level in those with resolved HBV infection. Five out of nine patients in our cohort exhibited a change in their ability to respond to moDC expansion and presented multiple epitopes against core, polymerase, and envelope after two years of TDF treatment. This was accompanied by changes in HBeAg seroconversion and decreased titers of HBsAg. In accordance with our findings, plasmacytoid DCs pulsed with HBV-derived peptides elicited a stronger anti-HBV immunity in HBeAg-negative patients than in HBeAg-positive patients [28]. Moreover, antiviral therapy may depress PD-L1 expression on myeloid DCs and restore T cell function [29]. In addition, our results demonstrated that other clinical parameters such as HBV viral load and genotype, and the patient’s alanine aminotransferase level did not correlate with the ability of chronic HBV patients to respond to moDC stimulation. Several peptides of HBV, including envelope (Env_183-191_), pre-S (Env_244-253_), core (Core_18-27_), and polymerase (Pol_575-583_) have been used in DC vaccines in patients [21, 30]. However, these peptide-pulsed DC immunotherapies depressed HBV replication, they had no effect on HBsAg levels. Identifying the optimal antigens for incorporation into a DC vaccine is crucial for HBV control. By analyzing the HBV-specific CD8^+^ T cell responses to overlapping epitopes in HBV infection at different stages, we identified several new immunodominant epitopes and found high immunoprevalence of T cell responses targeting Core_91-105_, Pol_796-810_, and Env_256-270_ in the TN group, Core_106-120_, Pol_376-390_, and Env_256-270_ in the TR group, and Core_16-30_, Pol_381-395_, and Env_256-270_ in the RS group. The immunodominance of the Env_256-270_ T cell response in comparison to other HBV-specific CTL responses can be explained by virological and immunological features. First, Env_256-270_ resides in the second transmembrane domain (TM2) region of the HBV envelope. TM2 is essential for the stable assembly of S chains by establishing intramembrane interactions. A mutagenesis approach indicated TM2 is required as a component of the type II signal in the S protein, which is crucial for the formation of stable multimers during subviral particle morphogenesis [31]. Studies have indicated high levels of conservation and infectivity in the transmembrane domains of the HBV envelope among various HBV genotypes [31, 32] and, hence, the frequent T cell response against it.

Many studies have shown that mutations in the HBV genome were significantly associated with disease pathogenesis and clinical outcome. HBV-specific CD8^+^ T cells play an important role in HBV control. Some studies have found that the T-cell response against Core_18-27_ was reduced in patients with aa mutation in the region of this core epitope [23]. Moreover, mutations in SHBs are able to block HBV surface protein synthesis and subviral particle production [26]. In current study, we found that a novel epitope comprising Env_256-270_ in the SHBs was immunoprevalent and immunodominant in HBV-infected subjects who were able to control HBV infection. To investigate whether mutations in this epitope affect its functional recognition by T cells, we performed HBV DNA sequencing and found only two types of amino acid substitutions, corresponding to the envelope region of Env_256-270_. We further demonstrated that Env_256-270_-specific CD8^+^ T cells were able to be activated by both variants of this epitope at residue positions of 258 and 261, corresponding to the mutations L95W and L98V in the SHBs. Therefore, the Env_256-270_ T cell response is an important component of HBV-specific adaptive T cell immunity in HBV-infected subjects. We also found that Env_256-270_ epitopes induced more responses in patients with HLA-A*02:03/02:06/02:07, while it seemed not to be able to induce a CD8^+^ T cell response in patients with HLA-A*02:01. This discordance may be due to the binding deficiency of HLA-A*02:01 with peptides or an alteration in the T-cell-receptor-recognition site. Whether these findings can be extrapolated to other epitopes and whether these responses are significantly involved in viral control clearly requires broader analyses with larger cohorts and a more diverse HLA background.

HBsAg clearance is correlated with higher PBMC responsiveness to HBV core_18-27_ in CHB patients receiving combined antiviral treatment with lamivudine and IFN-α [33]. In our study, the HBV-specific CD8^+^ T cell response against Env_256-270_ in the Peg-IFN treated subgroup of the RS group was comparable to that in the spontaneously resolved subgroup. This implies that the breakthrough of immune tolerance contributes to viral clearance. Unfortunately, serial blood samples were not collected at baseline or other time points during antiviral treatment for PBMC isolation to assess the HBV-specific CD8^+^ T cell response in the subgroup that received Peg-IFN treatment. Although we cannot assess the longitudinal change in the response before and after 48 weeks of Peg-IFN treatment, there was a trend for the antiviral treatment to improve immunologic reconstitution in chronic HBV infection.

Of note, our data do not address whether the CD8^+^ T cells specific for these epitopes stimulated by autologous moDCs have a protective or pathogenic role during HBV infection. However, the superior antiviral activity of vertical immunodominant epitopes has been demonstrated recently in a study that investigated the effect of HIV-specific CTL responses on HIV control and showed that vertical immunodominant epitopes exerted more pressure on HIV variability [34]. Therefore, the vertical immunodominance of epitopes on specific CD8^+^ T cells upon moDC expansion suggests the important antiviral activity of such a response during HBV infection.

In conclusion, we confirm the ability of expanded autologous moDCs to stimulate HBV-specific T cells and provide the profiles of HBV epitopes recognized by specific CTLs. Env_256-270_, corresponding to residues 93-107 in the SHBs, is a new T-cell-responding epitope in the HBV S protein outside the *“*a*”* domain, and it is associated with HBV-specific T cell immunity and disease stages. It might be a potential target epitope for DC-based immunotherapy for CHB patients with complete viral suppression by long-term NAs treatment.

## Electronic supplementary material

Below is the link to the electronic supplementary material.

Supplementary material 1 (PDF 484 kb)
