# Supplementary material for: A novel T-cell epitope in the transmembrane region of the hepatitis B virus envelope protein responds upon dendritic cell expansion
Source: Arch Virol. 2018 Nov 10;164(2):483–95. doi: 10.1007/s00705-018-4095-0 (PMC6373280; doi:10.1007/s00705-018-4095-0)
Supplement: Supplementary file 1 — Supplementary material 1 (PDF 484 kb) [file 705_2018_4095_MOESM1_ESM.pdf]

A novel T-cell epitope in the hepatitis B virus envelope transmembrane region response upon dendritic cell expansion

Lubiao Chen[1*], Ying Zhang[1*], Shaoquan Zhang[1], Youming Chen[1], Xin Shu[1], Jing Lai[1], Hong Cao[1], Yifan Lian[2], Zania Stamataki[3], Yuehua Huang[1, 2**]

[1] Department of Infectious Diseases, The Third Affiliated Hospital of Sun Yat-sen University, Guangzhou, China.

[2] Guangdong Provincial Key Laboratory of Liver Disease Research, The Third Affiliated Hospital of Sun Yat-sen University, Guangzhou, China.

[3] National Institute for Health Research Birmingham Liver Biomedical Research Unit, Institute of Immunology and Immunotherapy, University of Birmingham, Birmingham, United Kingdom.

[*]These authors contributed equally to this work.

[**]Corresponding author: huangyh53@mail.sysu.edu.cn

Correspondence:

Yuehua Huang, M.D., Ph.D.

Department of Infectious Diseases & Guangdong Provincial Key Laboratory of Liver Disease Research, The Third Affiliated Hospital of Sun Yat-sen University

600 Tianhe Road. Guangzhou, China, 510630

Tel: 8620-85252702

Fax: 8620-85253305

Email: huangyh53@mail.sysu.edu.cn

Materials and Methods

Clinical, virological and serological parameters

Liver function tests were measured using a Hitachi 7180 automatic analyzer (Hitachi Corporation, Japan). HBV genotype was determined by direct sequencing (ABI 3730, Applied Biosystem Inc., MA, USA). The S gene of HBV DNA was amplified by nested PCR and amplicons were submitted for direct sequencing. Serum sample was tested for anti-HBs, HBeAg, and anti-HBe using commercial kits (Abbott, IL, USA). HBsAg was quantified by the Elecsys HBsAg Ⅱ Quant reagent kits (Roche, IN, USA) according to the manufacturer's instructions (lower limit of detection 0.05 IU/ml). Serum HBV DNA level was measured by the Roche COBAS Ampliprep/COBAS TaqMan HBV test v2.0 (lower limit of detection 20 IU/ml, Roche, NJ, USA). Liver stiffness was measured to define the levels of liver fibrosis by FibroScan® (Echosens, Paris, France).

PBMC isolation and flow cytometry sorting

Peripheral blood mononuclear cells (PBMCs) were isolated by Ficoll density gradient separation and cryopreserved in liquid nitrogen. PBMCs were stained with anti-HLA-DR-PB (Invitrogen, CA, USA), anti-CD3-PerCP-Cy5.5 (BD Biosciences, CA, USA), anti-CD4-FITC (Invitrogen, CA, USA), and anti-CD8-PE-Cy7 (BD Biosciences, CA, USA) for 30 minutes at 4℃. Viability dyes, DAPI, or Live/Dead Yellow Fixable Stain (Invitrogen, CA, USA) were used to gate live cells followed by singlet gates. Cells were sorted by flow cytometry on BD FACSCanto (Becton Dickinson, CA, USA) to greater than 99% purity. Data from the sorting files were analyzed by BD FACSDiva software (Becton Dickinson, CA, USA) and used to calculate the frequency of APC populations in all subjects.

HBsAg-pulsed PBMCs or autologous moDC expansion

PBMCs (1-2×10^9 cells) were cultured in RPMI 1640 medium containing streptomycin and penicillin (Gibco, NY, USA). For preparing HBsAg-pulsed PBMC, 20% of PBMCs were loaded with HBsAg (3μg/ml) (BioKangTai, Shenzhen, China) and incubated for 1 hour at 37°C. HBsAg-pulsed PBMCs were washed and mixed back with remaining PBMCs. For HBsAg-pulsed moDC, autologous moDCs were prepared according to the method of Romani [1]. PBMCs were allowed to adhere on a plastic surface for 4 hours. Nonadherent cells were removed by gentle washing and saved in -80°C. The adherent cells were cultured at 37°C in RPMI 1640 containing 1% autologous plasma and antibiotics, and supplemented with granulocyte-macrophage colony-stimulating factor

(1000 U/ml) and interleukin-4 (500 U/ml) (R&D system, Abingdon, UK). Immature moDCs were harvested on day 6 and pulsed with HBsAg (3μg/ml) (BioKangTai, Shenzhen, China) for 4 hours, before undergoing a maturation step in culture medium containing tumor necrosis factor-alpha (20 ng/ml) (PeproTech, CT, USA). The moDCs were mixed with above nonadherent cells. Cell mixtures from both groups of "PBMC only" and "PBMC+DC" were further expanded in vitro for 10 days in Aim-V + 2% human AB serum + 20U/ml IL-2 (PeproTech, CT, USA), respectively. After 10-days expansion, cells were stimulated with the pool of overlapping 15-mer peptides and HBV-specific T cells were quantified using IFN-γ ELISPOT assay.

Autologous moDC phenotyping

Mature moDCs were surface-stained with monoclonal antibodies against human MHC-Ⅰ (Dako, CA, USA), CD11c (Beckman Coulter, CA, USA), CD80 (eBioscience, CA, USA), CD83 (eBioscience, CA, USA), and CD86 (Dako, CA, USA). After washing, they were incubated for 1 hour with the appropriate fluorescein isothiocyanate-conjugated secondary antibodies. Cells were washed again, and fixed in phosphate-buffered saline (PBS) containing 1% paraformaldehyde before flow cytometry sorting.

Supplementary Table 1 HLA-A2-resticted HBV epitopes in the tetramer detection

Peptide	Amino acid sequence	
	Genotype B	Genotype C
Envelope		
Env$_{194-202}$	*FLLTKILTI*	*FLLTRILTI*
Env$_{346-354}$	*WLSLLVPFV*	*WLSLLVPFV*
Env$_{349-358}$	*LLVPFVQWFV*	*LLVPFVQWFV*
Env$_{359-368}$	*GLSPTVWLSV*	*GLSPTVWLSV*
Polymerase		
Pol$_{453-461}$	*GLSRYVARL*	*GLPRYVARL*
Core		
Core$_{18-27}$	*FLPSDFFPSI*	*FLPSDFFPSI*

Underlined capitals indicate amino acid substitutions in different HBV genotypes.

Supplementary Table 2 Clinical and virological features of chronic hepatitis B patients for *ex vivo* dendritic cells differentiation

Characteristics	TN (n=40)	TR (n=40)	*P* value
Male gender[a]	30 (75%)	27 (68%)	NS
Age (years)[b]	28 (22-31)	33 (30-40)	NS
HBeAg (+)[a]	40 (100%)	0 (0%)	< 0.0001[*]
anti-HBe (+)[a]	0 (0%)	27 (68%)	< 0.0001[*]
ALT level (U/L)[b]	40 (31-148)	32 (25-39)	0.0475[*]
HBV DNA level ($\log_{10}$ IU/ml)[b]	7.06 (5.12-8.13)	ND	< 0.0001[*]
HBsAg level ($\log_{10}$ IU/ml)[b]	4.68 (3.65-5.09)	3.01 (2.69-4.21)	0.0116[*]
Genotypes[a]			
B	22 (55%)	25 (63%)	NS
C	18 (45%)	15 (37%)	—
FibroScan (kPa)[b]	5.8 (4.7-6.8)	6.1 (5.9-7.8)	NS

Abbreviations: HBeAg, hepatitis B e antigen; anti-HBe, hepaitits B e antibody; ALT, alanine aminotransferase; HBV, hepatitis B virus; HBsAg, hepatitis B surface antigen. ND, not detectable (HBV DNA < 20 IU/ml); NS, not significant ($P \geq 0.05$).

[a] Data were expressed as frequency (percentage).

[b] Data were expressed as median (interquatile range).

[*] Statistically significant ($P < 0.05$).

Supplementary Table 3 Clinical and virological features of nine CHB patients before and after treatment with tenofovir, for analysis of vertical immunodominance upon moDC differentiation *ex vivo*

Characteristics	Baseline	Week 96	*P* value
Age (years)[b]	30 (17-42)	—	—
Male gender[a]	8 (9%)	—	—
HBeAg (+)[a]	9 (100%)	7(78%)	NS
anti-HBe (+)[a]	0 (100%)	2(22%)	NS
ALT (U/L) [b]	99 (47-230)	33 (20-45)	0.0323[*]
HBV DNA (log_{10} IU/ml) [b]	7.1(4.9-8.6)	ND	< 0.0001[*]
HBsAg (log_{10} IU/ml) [b]	4.26 (3.7-5.1)	3.27 (2.1-4.7)	0.0112[*]
Genotype B[a]	9 (100%)	—	—
FibroScan (kPa) [b]	5.8 (4.8-7.5)	5.7(4.6-7.9)	NS

Abbreviations: HBeAg, hepatitis B e antigen; anti-HBe, hepaitits B e antibody; ALT, alanine aminotransferase; HBV, hepatitis B virus; HBsAg, hepatitis B surface antigen. ND, not detectable (HBV DNA < 20 IU/ml); NS, not significant.

[a] Data were expressed as frequency (percentage).

[b] Data were expressed as median (interquatile range).

[*] Statistically significant ($P < 0.05$).

Supplementary Table 4 Patient characteristics for the study of $Env_{256\text{-}270}$-specific CD8+ T cell responses

Characteristics	TN	TR	RS		*P* value	
	(n=20)	(n=20)	(n=20)	TN *vs.*TR	TN *vs.* RS	TR *vs.* RS
Male gender[a]	14 (70%)	16 (80%)	15 (75%)	NS	NS	NS
Age (y.o.)[b]	24(20-32)	36(30-40)	27(23-35)	NS	NS	NS
HBeAg (+)[a]	20 (100%)	0 (0%)	0 (100%)	< 0.0001[*]	< 0.0001[*]	NA
anti-HBe (+)[a]	0 (100%)	18 (90%)	20 (100%)	< 0.0001[*]	< 0.0001[*]	NS
ALT (U/L)[b]	72(20-101)	30(25-36)	27(22-38)	0.0413[*]	0.0380[*]	NS
HBV DNA (log_{10} IU/ml)[b]	7.0(5.0-8.5)	ND	ND	< 0.0001[*]	< 0.0001[*]	NA
HBsAg (log_{10} IU/ml)[b]	4.8(4.1-5.1)	3.0(2.7-4.1)	ND	0.0133[*]	< 0.0001[*]	< 0.0001[*]
Genotypes[a]						
B	13 (65%)	12 (60%)	15 (75%)	NS	NS	NS
C	7 (35%)	8 (40%)	5 (25%)	—	—	—
Others	0 (0%)	0 (0%)	0 (0%)	—	—	—
FibroScan (kPa)[b]	4.3(4.9-6.7)	5.2(5.9-7.8)	4.9(4.4-5.7)	NS	NS	NS

Abbreviations: HBeAg, hepatitis B e antigen; anti-HBe, hepaitits B e antibody; ALT, alanine aminotransferase; HBV, hepatitis B virus; HBsAg, hepatitis B surface antigen. ND, not detectable (HBsAg < 0.05 IU/ml; HBV DNA < 20 IU/ml); NA, not applicable; NS, not significant ($P \geq 0.05$).

[a] Data were expressed as frequency (percentage).

[b] Data were expressed as median (interquatile range).

[*] Statistically significant ($P < 0.05$).

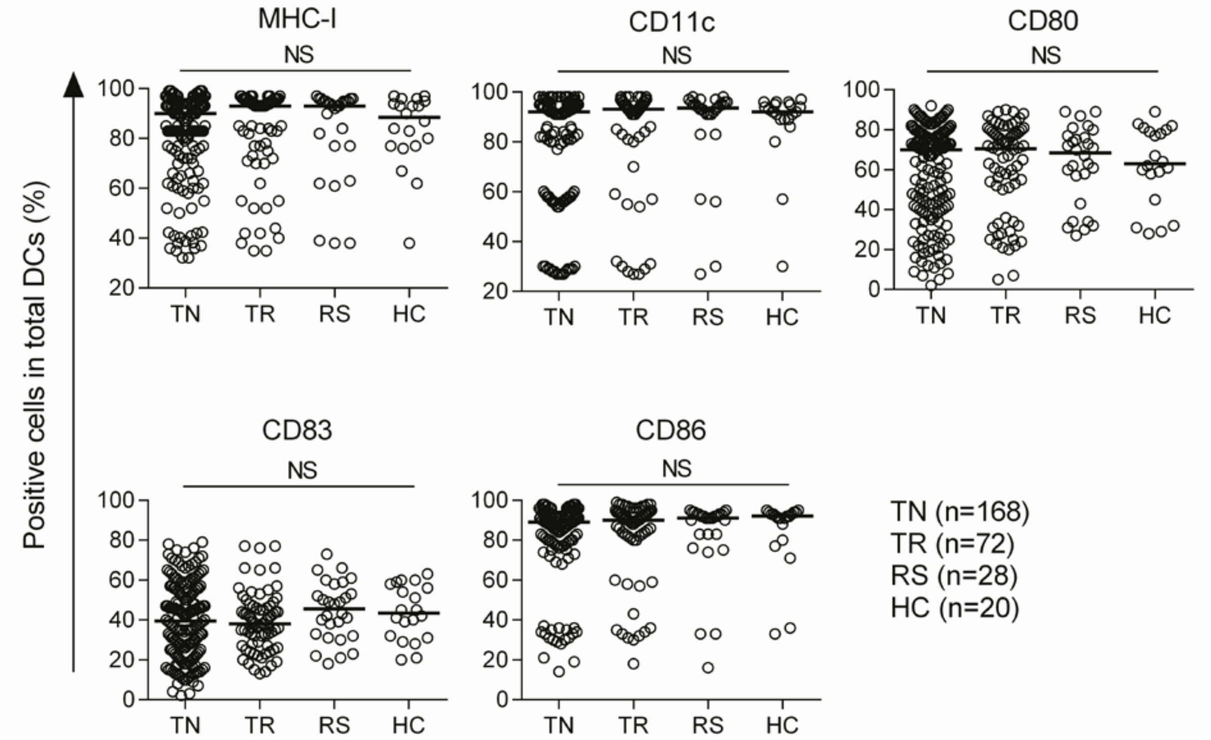

Supplementary Fig. 1 Surface markers on moDC populations in different patient groups and healthy control. Data are expressed as scatter plots and full lines indicate the medians. There are no significant differences in the proportions of positive cells in total moDCs regarding MHC-Ⅰ, CD11c, CD80, CD83, or CD86 sorting among the TN, TR, RS and HC groups. Kruskal-Wallis H test. NS, not significant ($P > 0.05$). moDCs, Monocyte-derived dendritic cells.

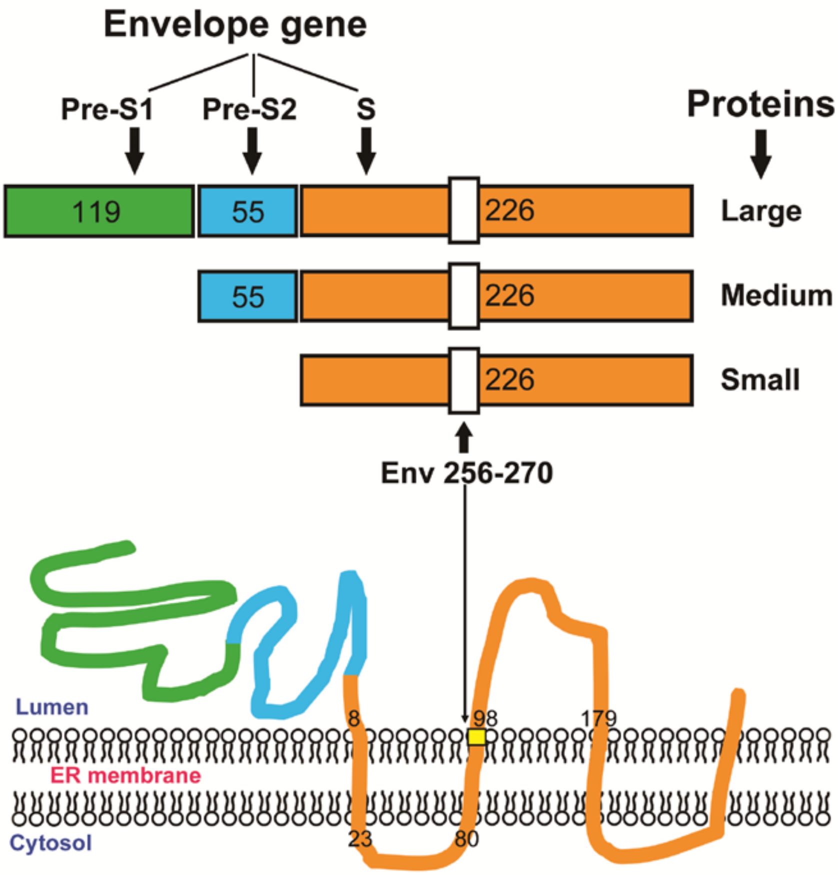

Supplementary Fig. 2 A schematic figure of HBV envelope gene and its translation products. The white rectangle represents the location of $Env_{256\text{-}270}$ amino acid sequence in small HBV surface protein (SHBs) and yellow rectangle indicates the location of amino acid residues corresponding to $Env_{256\text{-}270}$ within the second transmembrane domain of SHBs.

Reference

1. Schuler G, Brang D, Romani N (1995) Production and properties of large numbers of dendritic cells from human blood. Adv Exp Med Biol 378:43-52
